# Levothyroxine, mental confusion and suicide attempt

**DOI:** 10.1590/S1516-31802009000500013

**Published:** 2010-02-03

**Authors:** Amilton dos Santos, Neury José Botega

**Affiliations:** I MD. Psychiatrist, Faculdade de Ciências Médicas da Universidade Estadual de Campinas (FCM-Unicamp), Campinas, São Paulo, Brazil.; II MD. Head professor of psychiatry, Department of Medical Psychology and Psychiatry, Faculdade de Ciências Médicas da Universidade Estadual de Campinas (FCM-Unicamp), Campinas, São Paulo, Brazil.

In a general hospital, the risk of suicide among inpatients has been estimated to be three times greater than among the rest of the population.^1^ The most common method of suicide in these cases is jumping (from the hospital’s windows or balconies).^2^ Patients with psychiatric comorbidities (between 20 and 50%) are a group presenting considerable risk in clinical and surgical wards, particularly those with acute confusion or delirious states.^3^ In addition to psychiatric disorders due to organic illnesses, many medical procedures can also cause mental symptoms and lead to suicidal behavior, such as hormone therapy, other drug therapy, endocrine-metabolic responses to major surgery and azygoportal disconnection, among others.

A 38-year-old male farm worker, with a background of incomplete elementary education, was admitted to the adult general ward of the Clinical Hospital of the State University of Campinas (Unicamp), diagnosed with myxedematous myopathy, which led to the following clinical sequence: rhabdomyolysis, myoglobinuria and acute renal failure.

His clinical treatment had started three years earlier, with a diagnosis of primary hyperthyroidism. He underwent three applications of radioiodine and, after the third one, developed hypothyroidism. Levothyroxine 100 mcg/day was prescribed, but he did not use the medication because he was unable to read and therefore did not understand the prescription. The medical team that provided his outpatient endocrine care was unaware he was not taking the hormone replacement medication.

In the ward, because it was assumed he had been in daily use of levothyroxine, his prescription was maintained. Two days after admission, although the renal failure had already been reversed, he woke up confused, disoriented, with psychomotor agitation and impaired judgment of reality, saying that he was being pursued and that people wanted to kill him. When a bed was brought to the corridor by nurses, he considered that it was an ambush. Desperate to flee, he threw himself from the 6^th^ floor (a drop of eight meters). The fall was partly contained by a protection net. Following this episode, the psychiatric disorder was reversed, after temporary introduction of haloperidol, with no new episodes. He was discharged with a prescription for 50 mcg/day of levothyroxine, without suicidal ideation. The additional diagnostic hypothesis of *delirium* was made. The iatrogenic levothyroxine poisoning had precipitated or exacerbated the confusion in this previously weakened patient, thereby leading to psychiatric symptoms that resulted in his jumping.

*Delirium* is a syndrome that occurs in approximately 10 to 30% of clinical inpatients. Its features include acute disturbance of the level of awareness and overall impairment of cognitive functions, attention, memory and orientation. There are also frequent abnormal perceptions, delusions, restlessness, agitation and disturbances of sleep and affection.^4^ This condition often remains undiagnosed. Physicians need to recognize it, to identify and treat the underlying causes and to prevent the development of its complications.

Besides clinical and psychiatric care for patients with confused and agitated states, several other measures are recommended when aiming to prevent suicide in a general hospital, such as: restriction of access to means of suicide (windows and hazardous balconies); placement of protective nets; training of the care team for early detection of and intervention in any mental disorders; and assessment of suicide risks before hospital discharge^5^.
